# Metalens and microtaper spectrometers on a fingertip

**DOI:** 10.1038/s41377-023-01217-z

**Published:** 2023-07-06

**Authors:** Peixia Zheng, Xuan Zhang, Hong-Chao Liu

**Affiliations:** Institute of Applied Physics and Materials Engineering, University of Macau, Avenida da Universidade, Taipa, Macao SAR China

**Keywords:** Metamaterials, Fibre optics and optical communications

## Abstract

A multi-foci metalens and a leaky-mode microtaper provide innovative platforms to achieve high-resolution, wideband light spectra in millimeter-sized devices, thereby paving new ways for the commercialization of on-fingertip spectrometers.

## Abstract (web summary)

A multi-foci metalens and a leaky-mode microtaper provide innovative platforms to achieve high-resolution, broadband and compact spectrometers.

## Graphical abstract

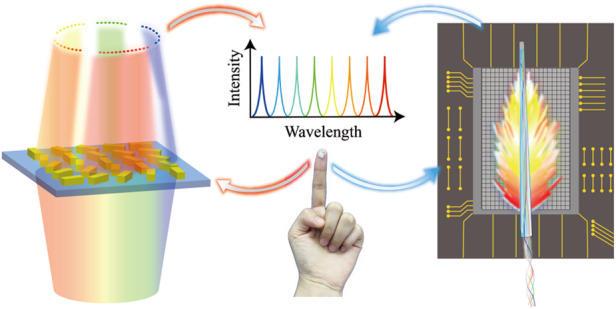

With the development of on-chip integrated photonics technology, researchers are exploring ways to create compact and portable spectrometers those can be easily integrated into various devices and systems. These miniaturized spectrometers are expected to perform highly precise measurements of light-matter interactions, enabling them to identify chemical compositions of different materials, detect pollutants in the environment, and diagnose medical conditions in real-time. This has led to the development of a variety of new applications for spectrometers from healthcare and biotechnology, such as wearable medical devices, to environmental monitoring and agriculture, such as handheld environmental detectors. Although the miniaturization of spectrometers remains lots of challenges, recent advancements in nanofabrication techniques and materials science have made it possible to fabricate powerful, low-cost, and compact spectrometers those are capable of delivering high-quality light spectra. As the demand for miniaturized spectrometers continues to grow, researchers are pushing the boundaries of their capabilities, paving the way for exciting new innovations in the field of optics and photonics.

Metasurfaces and microfibers exhibit immense potential in various optical applications due to their ultra-compact nature. As a typical kind of metasurfaces, metalenses can manipulate the dispersion of light at will and provide precise control over the path of light, making it an attractive candidate for imaging and optical communication. Meanwhile, microfibers can transmit and reflect light with minimal distortion, making them useful tools for spectral measurements. In two recent papers published in Light: Science & Applications and eLight, two research groups led by Heriot-Watt University and Zhejiang University proposed the use of multi-foci metalens and leaky-mode microtaper to obtain high-resolution spectral information, respectively.

In the paper entitled “Compact multi-foci metalens spectrometer”, Prof. Xianzhong Chen’s group proposed a multi-foci metalens that can focus light at multiple wavelengths^1^, as shown in the left part of Fig. 1. Multi-foci metalens usually have small planner structures which can simultaneously focus different colors of light into multiple spots, allowing for a single-shot spectroscopy and imaging. Although sub-regional design and folded metalenses have been proposed to achieve multi-wavelength dispersion control^2–5^, limited pixels designed for each wavelength always result in poor beam convergence quality or multiple focal points at different focal lengths. Creatively, the authors in this work demonstrate a compact metalens with multiple off-axis foci in the same focal plane with nearly the same spot size and maximum intensity, which perfectly and effectively solved this problem. Based on the intrinsic dispersion and multi-foci properties of a metalens device, the wavelength information is converted into the intensity distribution of different focal points on the same plane. Experimental results demonstrate that the proposed compact metalens spectrometer can split and focus different wavelengths at 180 predesigned focal points under the illumination of both monochromatic and polychromatic light beams. It can achieve nanometer-level spectral resolution over a broadband wavelength in the visible domain at a working distance of 300 μm. With ease of fabrication, this metalens-based spectrometer technique exhibits great potential for on-chip integrated photonics and compact spectrometry applications, including chemical sensing and environmental monitoring.

**Fig. 1:**
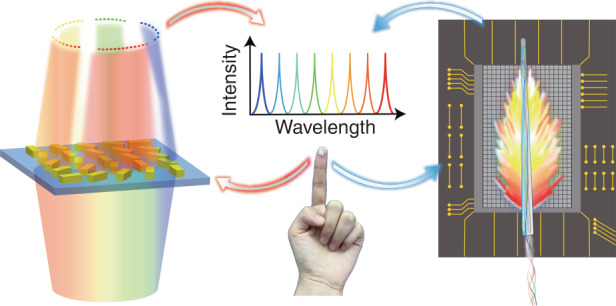
Schematic view of the metalens and microtaper spectrometers on a fingertip

In the other paper entitled “Microtaper leaky-mode spectrometer with picometer resolution”, the authors proposed a microtaper with enhanced leaky mode for spectral sensing^6^, as shown in the right part of Fig. 1 Microfibers are ideal small-size tools for manipulating light fields. Microtaper is a microfiber-based device that can force the guided modes to the leaky mode over a short distance if drawn under non-adiabatic conditions. Multimode interference in optical fibers has been proposed to generate random speckles associated with spectra information^7^, for example, a compact all-fiber speckle spectrometer was demonstrated by using cascading coreless fibers and photonic cryptal fibers^8^. However, conventional methods such as rough surface and multimode fiber usually require bulky or expensive detectors for speckle measurement, limiting their applications^9,10^. Moreover, leaky modes are generally considered undesirable in previous works. Creatively, Prof. Yaoguang Ma’s group proposed to use leaky modes of a microtaper as a means of spectral information retrieval. It is known that leaky modes can be generated by adjusting the fiber geometry. The authors constructed a scalable spectrometer system that combined a curved microfiber taper tip, a complementary metal-oxide semiconductor (CMOS) imaging sensor, and a light-weight vision transformer (ViT) network to achieve hyperspectral imaging^6^. In the system, the microfiber taper tip can generate complex leaky modes speckle as a result of the coupling between different modes introduced by the fiber geometry. Then, data acquisition is completed by a CMOS imaging sensor thanks to the easy-to-manufacture and integrated fiber taper structure. The ViT network as the brain of the system, can finally identify correlations between spectral information and leaky mode images. When light passes through the microtaper, it excites a leaky mode that is highly sensitive to the incident light spectra. By measuring the speckles of the leaky mode, researchers can extract the spectral information of the input light field. This microtape spectrometer can operate within a wide range of wavelengths, from 450 nm to 1000 nm with a high resolution of 1 pm. Moreover, the core components cost less than $15, making it a low-cost solution for spectral sensing applications. The proposed flexible and stable system is expected to have numerous applications in various fields, such as food inspection, drug identification, personalized health diagnostics, etc.

Both two works above show the utilization of ultra-compact components, with one metalens of 300 × 300 μm^2^ while the other focusing on microtapers of 1 mm^2^, both of which construct high-resolution spectrometers. They demonstrate the versatility and novel potential of metalens and microtapers in spectral sensing and analysis. While both works contribute to the miniaturization development of spectrometers, they differ in their approaches to spectral analysis. In Ref. ^1^, the metalens is a direct spectrometer that splits and focuses multiple wavelength light into predesigned focal points on the same plane, allowing for the direct reading of different wavelength field distribution. While in Ref. ^6^, the microtaper serves as the leaky mode generator to give different speckles according to different spectral information. Later, a computational process is required to obtain the final spectra. Two proposed spectrometers in both two works exhibit good flexibility, stability, and resolution, making them potential candidates for on-fingertip spectrometers, especially for the measurements of line spectra. However, the importance of continuous spectra cannot be overlooked, and further studies are expected to explore their potential for different kinds of spectra retrieval. Overall, both two works broaden the miniaturization of spectrometers, provide novel ways to increase resolution and working bandwidth, and steer the development of emerging spectrometers.
